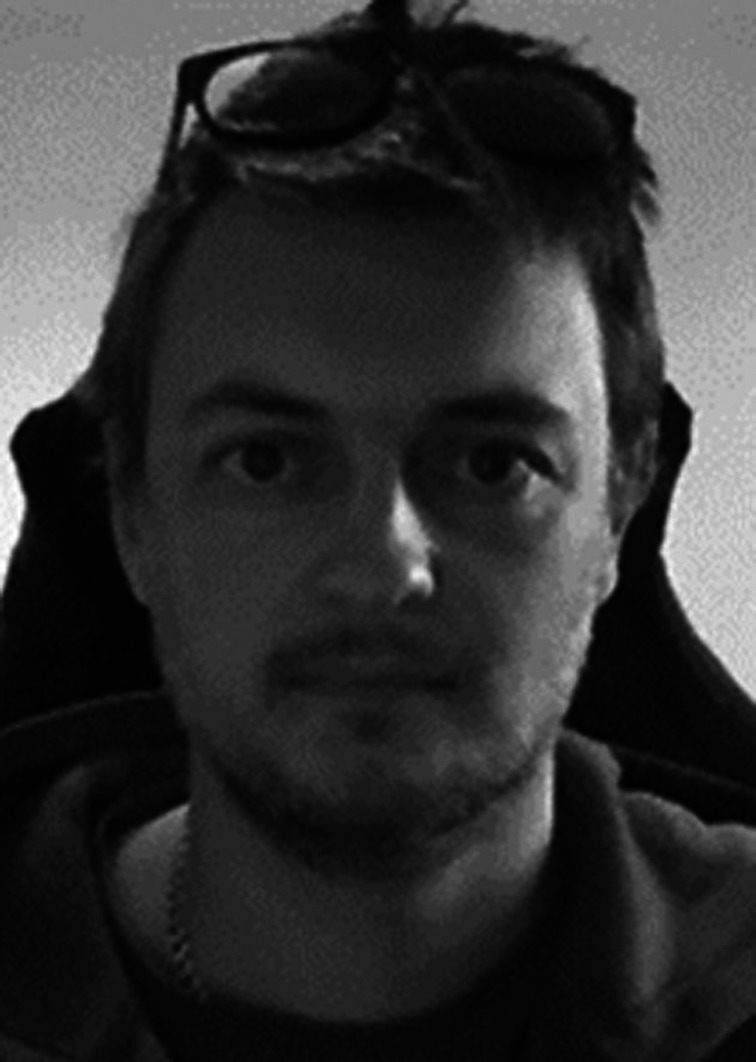# Mechanochemical Organocatalysis: Do High Enantioselectivities Contradict What We Might Expect?

**DOI:** 10.1002/cssc.202102157

**Published:** 2021-12-09

**Authors:** Matthew T. J. Williams, Louis C. Morrill, Duncan L. Browne

**Affiliations:** ^1^ Cardiff Catalysis Institute School of Chemistry Cardiff University Park Place Cardiff CF10 3AT UK; ^2^ Department of Pharmaceutical and Biological Chemistry School of Pharmacy University College London 29–39 Brunswick Square, Bloomsbury London WC1N 1AX UK

**Keywords:** ball-milling, catalysis, enantioselectivity, mechanochemistry, organocatalysis

## Abstract

Ball mills input energy to samples by pulverising the contents of the jar. Each impact on the sample or wall of the jar results in an instantaneous transmission of energy in the form of a temperature and pressure increase (volume reduction). Conversely, enantioselective organocatalytic reactions proceed through perceived delicate and well‐organised transition states. Does there exist a dichotomy in the idea of enantioselective mechanochemical organocatalysis? This Review provides a survey of the literature reporting the combination of organocatalytic reactions with mechanochemical ball milling conditions. Where possible, direct comparisons of stirred in solution, stirred neat and ball milled processes are drawn with a particular focus on control of stereoselectivity.

## Graphical abstract

**Enantioselective mechanochemistry**? The ability to achieve high enantioselectivies via organocatalytic reactions using the technique of ball‐milling represents a dichotomy. The technique of milling imparts energy and mixing to the system through collisions between the ball, walls of the milling jar, and the substrate. Here, the literature is surveyed to draw some comparisons to solution‐ and neat‐stirred reaction techniques.

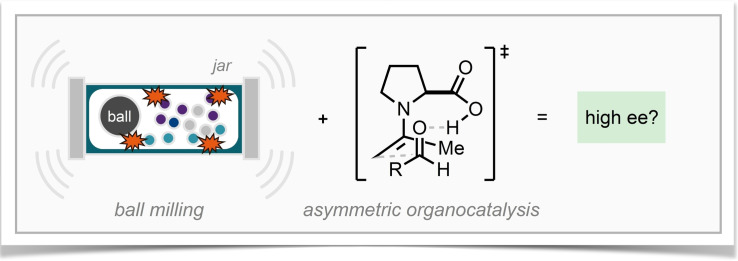

## 1 Introduction

Mechanochemical processes use the input of mechanical force to affect a reaction. Typically, applied forces are either opposing in direction and result in the application of pulling forces to a mechanophore (using sonication devices or atomic force microscopes) or the forces converge on the reaction centre through impact.[^1^, ^2^, ^3^, ^4^, ^5^, ^6^, ^7^, ^8^, ^9^] Such converging forces can be achieved with a mortar and pestle, a simple hammer, a diamond anvil cell or perhaps most typically by a ball mill.[^10^, ^11^, ^12^, ^13^, ^14^, ^15^, ^16^, ^17^, ^18^, ^19^, ^20^, ^21^, ^22^, ^23^, ^24^, ^25^, ^26^, ^27^, ^28^, ^29^, ^30^, ^31^] Ball‐milled reactions are conducted in jars using the displacement, movement and impact of balls to input energy into a sample. There are two key types of mills that are often used for synthetic chemistry. A planetary mill features a main horizontal wheel known as the sun wheel. Built on to this main wheel is a housing to secure the jars. When the sun wheel is rotated at the specified frequency the vertical jars rotate in a counter‐directional manner. Often, many small balls are loaded into a reactor of this type and the balls grind around the outside imparting friction or shear forces to the sample, before reaching a point in the revolution where they “pull‐off” from the wall and impact on the opposite side. The alternative type of mill, which is increasing in popularity for chemical synthesis applications, is a mixer mill. Mixer mills operate a little differently; the jars are mounted on motors in a horizontal position and are shaken by lateral displacement. Typically, the balls are larger than those used in a planetary reactor and only a single ball (perhaps two or three max.) is used. In contrast to the planetary mill, the lateral displacement of a mixer mill results in increased impact forces at the expense of frictional/shear forces. This can lead to increased latent heat generation. This type of reactor technology is very effective at homogenizing reactions mixtures (mixing) and inputting energy in the form of heat and pressure to reaction centres. All of this can be largely achieved in the absence of solvent, and thus the reactor environment of a ball mill is receiving increasing interest in recent times.[^32^, ^33^, ^34^, ^35^, ^36^, ^37^, ^38^, ^39^, ^40^, ^41^, ^42^, ^43^, ^44^, ^45^, ^46^, ^47^, ^48^, ^49^, ^50^, ^51^, ^52^, ^53^, ^54^, ^55^, ^56^, ^57^, ^58^, ^59^, ^60^]

**Figure 1:**
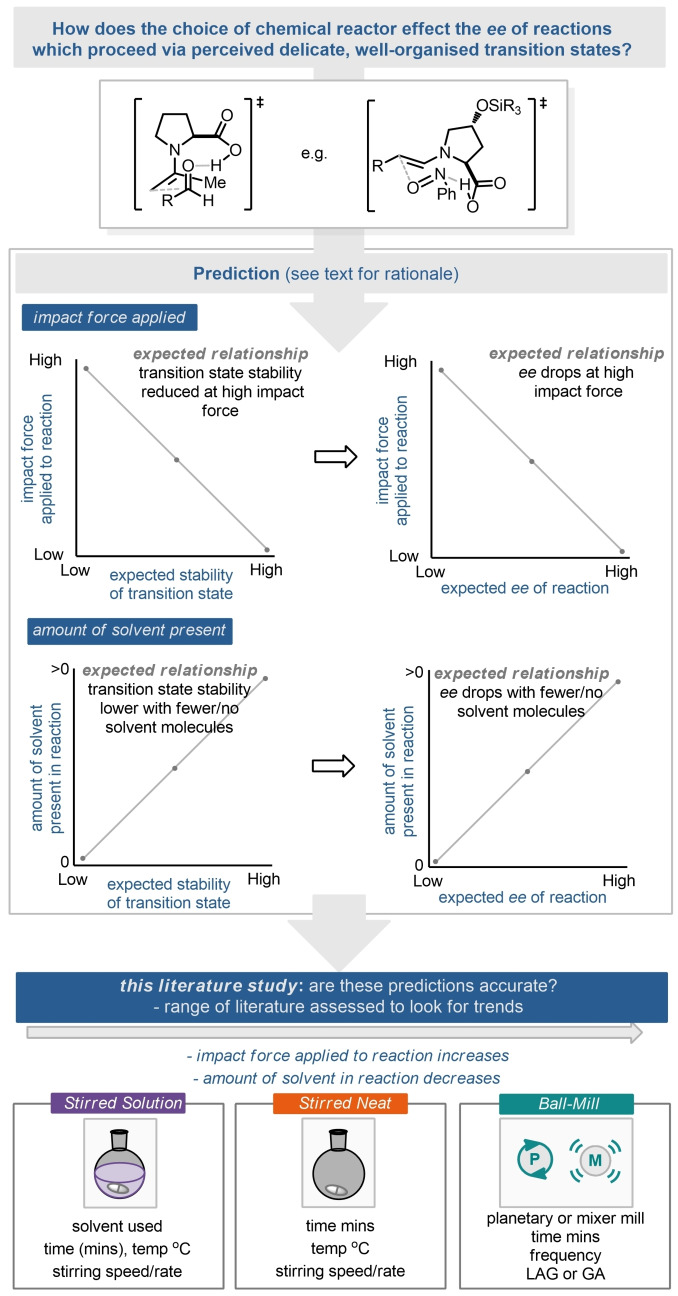
Would you expect high enantioselectivities from an organocatalytic reaction run in a ball mill in the absence of bulk‐solvent?

The use of ball mills has been studied for the preparation of co‐crystals,[^61^, ^62^, ^63^] unique polymorphs, zeolites,[^64^, ^65^] and metal‐organic frameworks (MOFs),^[66]^ for example. Applications to synthetic organic chemistry are also well known with several excellent early examples.[^67^, ^68^, ^69^, ^70^, ^71^, ^72^] However, in recent years the combination of this reactor technology with synthetic methods has realized many exciting solvent‐minimised protocols for metal‐catalysed processes (such as Pd,[^73^, ^74^, ^75^, ^76^, ^77^, ^78^, ^79^, ^80^, ^81^, ^82^, ^83^, ^84^, ^85^, ^86^, ^87^, ^88^, ^89^, ^90^, ^91^, ^92^] Rh,[^93^, ^94^, ^95^, ^96^, ^97^, ^98^] Ni),[^99^, ^100^, ^101^] the preparation and use of organometallics as well as delivering concepts such as piezoelectric‐driven radical reactions.[^102^, ^103^, ^104^, ^105^, ^106^, ^107^] An interesting area to combine with mechanochemistry is organocatalysis, which is commonly viewed as a green technique in its own right, due to being metal‐free and having the potential to be conducted in aqueous media.[^108^, ^109^, ^110^, ^111^, ^112^, ^113^, ^114^, ^115^, ^116^] Therefore, there has been considerable effort to combine the areas of mechanochemistry and organocatalysis to access a metal‐ and solvent‐free synthetic method capable of achieving good enantioselectivity for C−C and C‐heteroatom bond formation. Whilst other comprehensive Reviews on mechanochemical organocatalysis exist, including the use of other enabling technologies and reactor types, we are intrigued by the idea that high enantiomeric excesses (*ee* values) can be afforded by a reactor technology that essentially pulverises the input samples.[^23^, ^117^, ^118^, ^119^, ^120^, ^121^, ^122^] How can the perceived delicate and relatively complex transition states of organocatalytic reactions hold up under high impact (Figure 1)? At first glance we would predict that as the impact force increases on a transition state the stability of the transition state and resulting *ee* of the product would decrease. Conversely, we would predict that the stability of a transition state might also decrease in the absence of supporting solvent. Herein, we present curated literature data to investigate these predictions. The literature data are summarised firstly by an overview of the reported milling process, which is then followed by a specific reaction where we have a direct comparator of reactor types; stirred solution, stirred neat and ball milled (Figure 1). The corresponding parameters for each reactor type are also listed, including solvent, reaction time, catalyst loading, reaction temperature, stirring speed (where given) and, specifically for ball milling, the type of mill, milling frequency and note of any liquid assisted grinding (LAG) agents[^26^, ^34^, ^80^] or grinding auxiliaries (GA).

## 2 Secondary Amine Organocatalysis

### 2.1 Aldol reactions under ball‐milling conditions

**Scheme 1:**
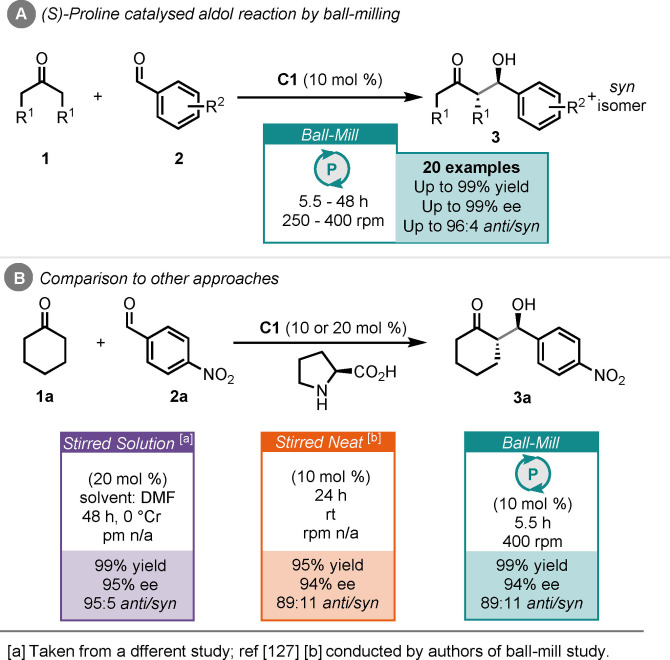
(A) (S)‐proline catalysed aldol reaction by ball‐milling. (B) Comparison to other approaches.

The aldol reaction is an established C−C bond forming transformation. Pioneering work on organocatalytic versions of this reaction were carried out by Hajos and Parrish,^[123]^ then later by Barbas and co‐workers,^[124]^ utilising (*S*)‐proline as a secondary amine organocatalyst. Most recently List and MacMillan were awarded the 2021 Nobel Prize in Chemistry “for the development of asymmetric organocatalysis”. Exploring the combination of organocatalysis with ball mills, Bolm and co‐workers investigated the (*S*)‐proline‐catalysed aldol reaction between ketones (**1**) and aromatic aldehydes (**2**) (Scheme 1).[^125^, ^126^] They found that using 10 mol % of (*S*)‐proline (**C1**) in a ball‐mill with rotation speeds between 250–400 rpm could produce the corresponding aldol products in up to 99 % yield, 99 % ee and 96 : 4 *anti*/*syn* ratio (Scheme 1A). The milling cycles in these reactions included pauses, to prevent overheating of the reaction mixture in the milling jars. In the specific case of reacting cyclohexanone (**1** **a**) with *p*‐nitrobenzaldehyde (**2** **a**), the product (**3** **a**) was obtained in 99 % yield, 94 % ee and 89 : 11 *anti*/*syn* after 5.5 h at 400 rpm. They carried out a stirred neat comparison, which highlighted that 24 h reaction time was required to match the performance in the ball‐mill. An alternative report demonstrates that with stirred solvent conditions (DMF) at 0 °C 48 h reaction time is required to afford complete conversion and a comparable ee to that of the ball‐milled and stirred neat process. Notably, the diastereoselectivity in the solvent process is greater in solvent than in the absence (95 : 5 in solvent vs. 89 : 11 in absence).^[127]^ This landmark report by Bolm and co‐workers established that solvent‐free organocatalytic reactions can be carried out in a ball mill, without sacrificing yield or enantioselectivity.

**Scheme 2:**
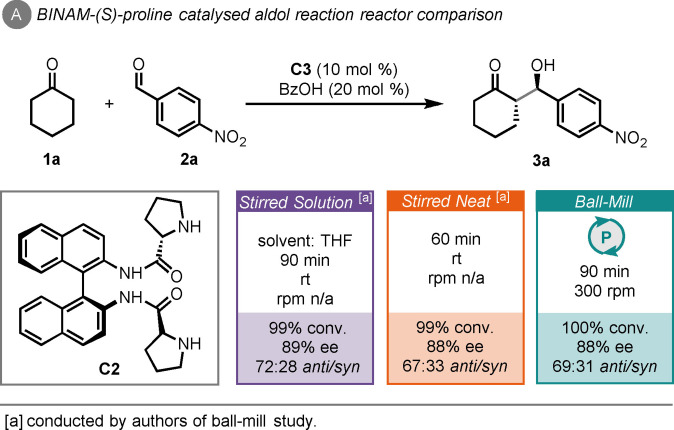
BINAM‐(S)‐proline‐catalysed aldol reaction of cyclohexanone and *p*‐nitrobenzaldehyde and comparison to other approaches.

Subsequently, Viózquez and co‐workers showed that the aldol reaction between cyclohexanone (**1** **a**) and *p*‐nitrobenzaldehyde (**2** **a**) could be catalysed by a BINAM‐(*S*)‐proline catalyst (**C2**) and benzoic acid as an additive, giving 100 % conversion to the aldol product (**3** **a**) in 1.5 h of ball‐milling (Scheme 2A).^[128]^ Moderate stereocontrol was achieved (88 % *ee* and 69 : 31 *anti/syn*). In comparison to stirred solution (THF) and stirred neat, there were moderate time savings in the latter reaction mode (60 vs. 90 min) but *ee* (88 or 89 %) and diastereomeric ratio (d.r.≈7 : 3) remained consistent with this catalyst.

**Scheme 3:**
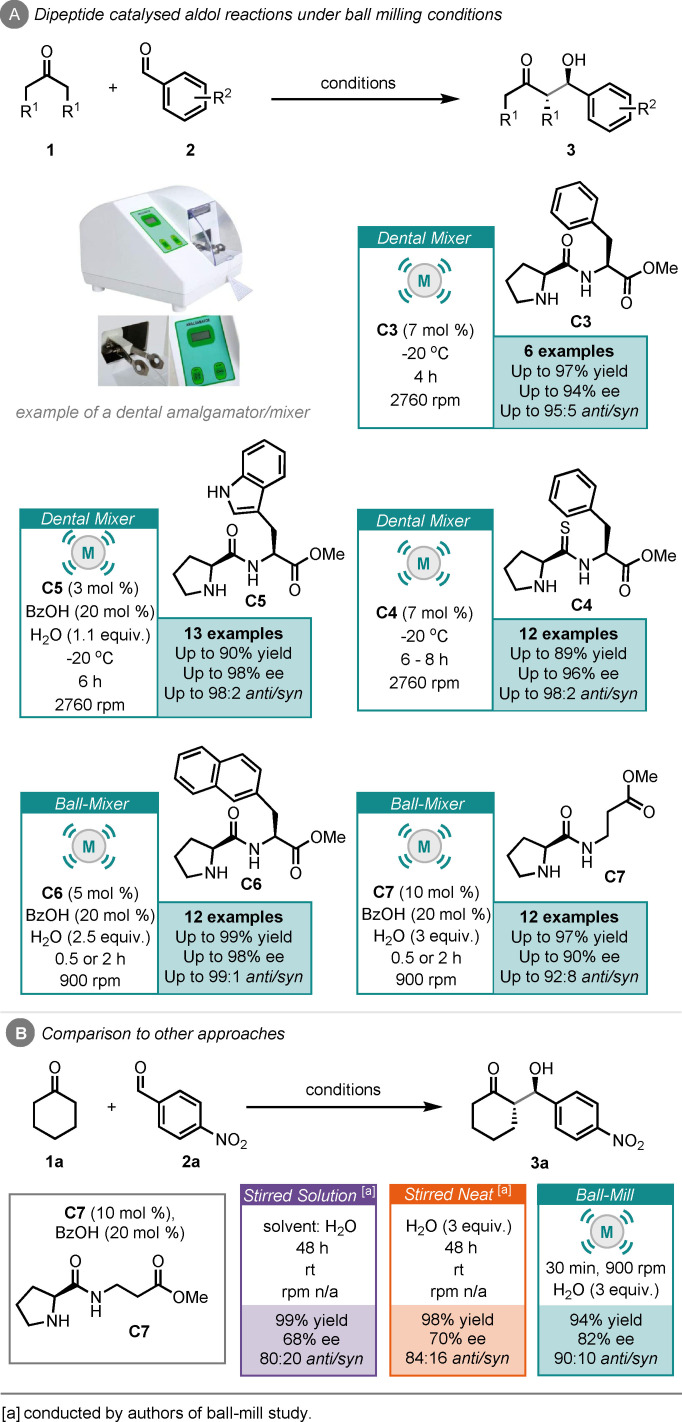
(A) A series of reports on the dipeptide (**C3**–**C7**)‐catalysed aldol reaction by ball‐milling. (B) Comparison to other approaches.

Juaristi and co‐workers then published five separate reports of dipeptide‐catalysed aldol reactions under ball‐milling‐type conditions (Scheme 3).[^129^, ^130^, ^131^, ^132^, ^133^] Notably three of the five reports creatively use a dental amalgamator (used in dentistry to prepare amalgams prior to treatment of cavities) to achieve the milled reaction results. The first of which featured the use of an (*S*)‐proline‐(*S*)‐phenylalanine dipeptide catalyst (**C3**), achieving high yields and stereoselectivities in as little as 4 h (Scheme 3A).^[129]^ Their subsequent reports focused on modifying this dipeptide catalyst to improve the performance, with each change having a specific purpose. These modifications included using tryptophan as the second amino acid residue (**C5**) to improve the lipophilicity of the system, which based on their proposed transition state, would help repel water molecules (Scheme 3).^[130]^ Another modification featured the use of a naphthylalanine as the second amino acid residue (**C6**), to probe π‐π interactions between the catalyst and aromatic ring of the aldehyde starting material in the transition state. This was found to be particularly important for electron‐poor aldehydes, for example, *p*‐nitrobenzaldehyde, achieving excellent yields and stereoselectivities in as little as 30 min.^[131]^ Thiodipeptides (**C4**) were also tested, giving improved stereoselectivities over the dipeptide analogue, which was attributed to an increase in acidity of the thioamide N−H bond, thus improving interactions in the transition state.^[132]^ Finally, α,β‐dipeptide (**C7**) was evaluated, to probe whether the second stereogenic centre of the catalyst was necessary for the high stereoselectivities observed previously.^[133]^ Remarkably, they found that this α,β‐dipeptide catalyst was very efficient in the aldol reaction, giving *ee* values up to 90 % and up to 92 : 9 *anti*/*syn* ratio. They then carried out solvent (water) and neat/concentrated comparisons, using catalyst **C7**, finding that reaction times of 48 h were required to achieve comparable yields, although with lower stereoselectivity (Scheme 3B). This study appears to mark the first example where ball‐milling leads to improved enantioselectivity in an organocatalyzed reaction and also features the use of water as a LAG agent.^[26]^

### 2.2 Other secondary amine‐catalysed transformations under ball‐milling conditions

**Scheme 4:**
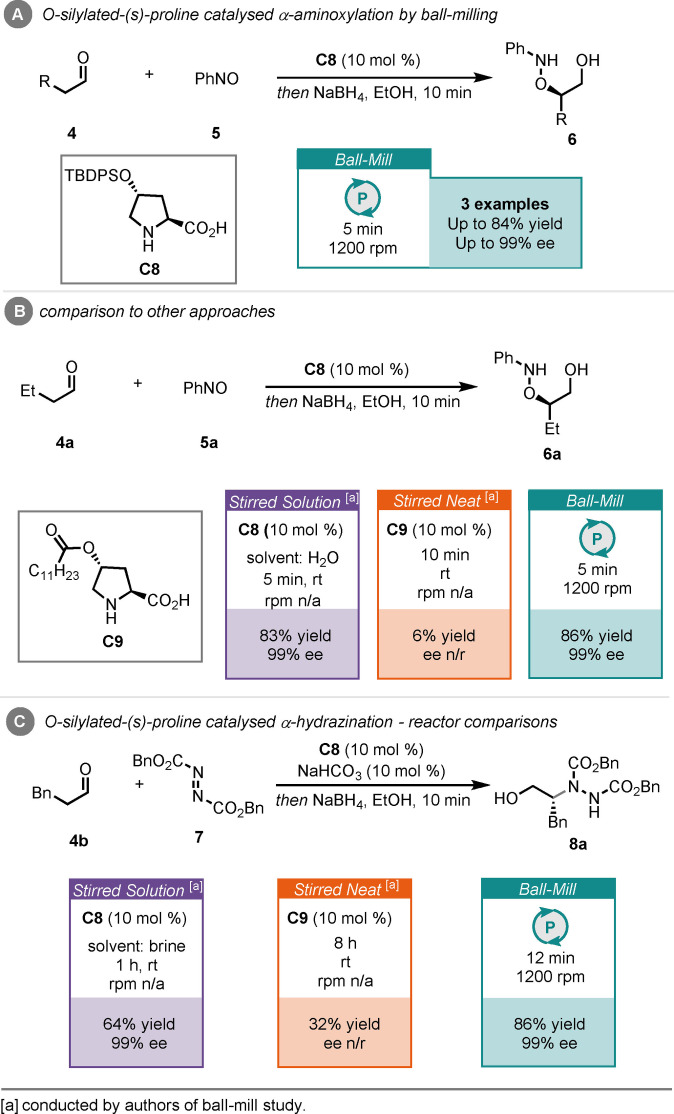
(A) Secondary amine‐catalysed α‐aminoxylation of aldehydes by ball‐milling. (B) Comparison to other approaches. (C) Secondary amine‐catalysed α‐hydrazination of aldehydes and comparison to other approaches.

Šebesta and co‐workers reported α‐aminoxylation and α‐hydrazination of aldehydes (**4**) with nitrosobenzene (**5**) and dibenzyl azodicarboxylate (**7**), respectively, catalysed by an *O*‐silylated‐(*S*)‐proline catalyst (**C8**), in a ball mill (Scheme 4A).^[134]^ For the α‐aminoxylation, high yields and enantioselectivities of the products (**6**) were obtained after 5 min of milling, followed by borohydride reduction for 10 min. Comparison reactions were also reported, revealing that the stirred solution (water) process could compete with the ball‐milling reaction; however, the stirred neat reaction was largely unsuccessful, giving a poor 6 % yield of product (Scheme 4B). This solvent‐free reaction used the alternative *O*‐acylated‐(*S*)‐proline catalyst **C9**. The α‐hydrazination of 3‐phenyl propanal (**4** **b**) was equally efficient under ball‐milling conditions, giving the product (**8** **a**) in 82 % yield and 99 % *ee*, after 12 min of milling, followed by borohydride reduction for 10 min. The solution comparison required a 1 h reaction time to achieve comparable enantiocontrol, albeit with a lower yield (Scheme 4C), whereas the solvent‐free reaction performed poorly, even after 8 h of reaction time.

### 2.3 Michael additions under ball‐milling conditions

**Scheme 5:**
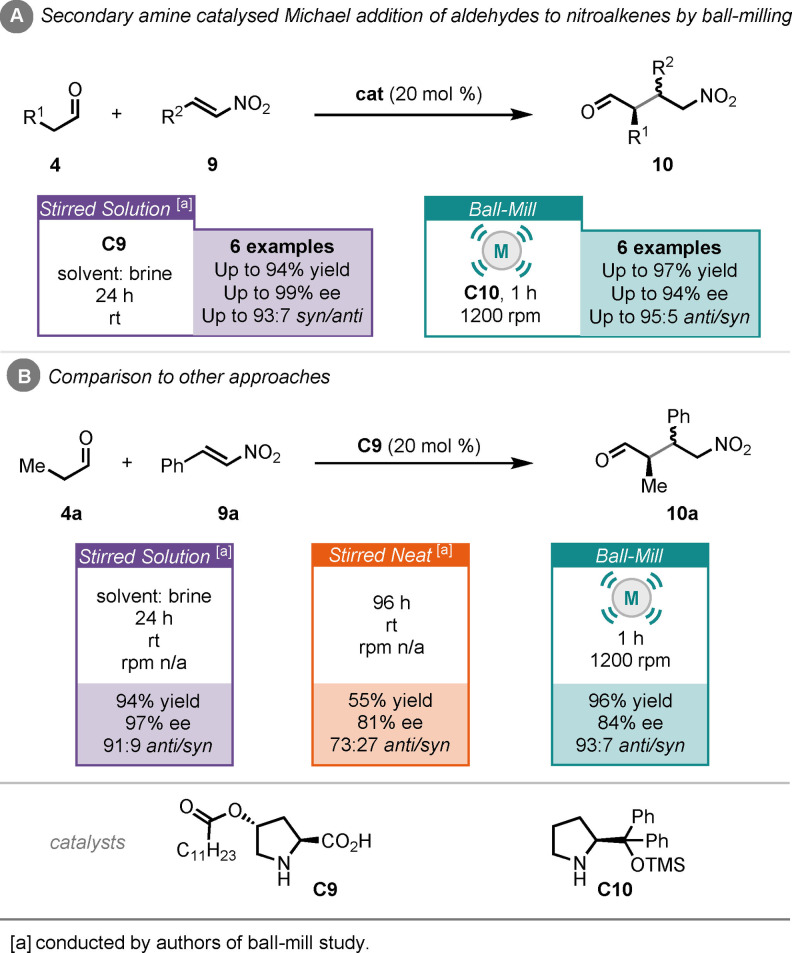
(A) Secondary amine‐catalysed Michael addition of aldehydes to nitroalkenes by ball‐milling. (B) Comparison of ball‐milling to other approaches.

Michael/1,4‐addition reactions are another powerful transformation that can be mediated by organocatalysts and are well established in solution chemistry.^[112]^ Šebesta and co‐workers investigated Michael additions under ball‐milling conditions. Their work involved Michael addition between enolisable aldehydes (**4**) to nitroalkenes (**9**), catalysed by Jørgensen‐Hayashi secondary amine (**C10**), under ball‐milling conditions (Scheme 5A).^[135]^ Reaction times as short as 1 h were achieved, giving products (**10**) in up to 97 % yield, 94 % *ee* and 95 : 5 *syn*/*anti* ratio. Specifically, for the reaction between propionaldehyde (**4** **a**) and β‐nitrostyrene (**9** **a**) catalysed by modified proline catalyst **C9**, the authors explored both stirred in solvent (brine) and stirred neat comparisons (Scheme 5B). For the milled example a yield of 96 % was obtained in 1 h with 84 % *ee* and 93 : 7 d.r. It was found that for the neat reaction a reaction time of 96 h was required to produce the Michael addition product (**10** **a**) in 55 % yield, 81 % *ee* and 73 : 27 d.r. On the other hand, a reaction time of 24 h was required for the solution reaction conditions to yield product in high yield (94 %) and high stereoselectivity (97 % *ee* and 91 : 9 d.r.). These comparisons reveal that the stirred neat process is inferior in all aspects to either the ball‐milled or solvent‐based approaches.

#### 2.3.1 Hydrogen‐bonding‐mediated additions under ball‐milling conditions

**Scheme 6:**
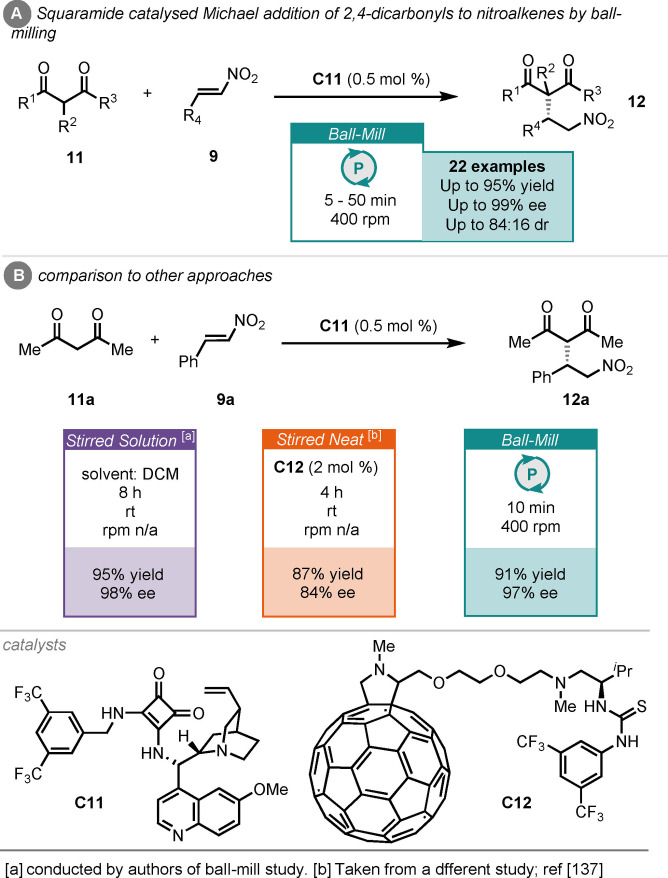
(A) Squaramide‐catalysed Michael addition of aldehydes to nitroalkenes by ball‐milling. (B) Comparison to other approaches.

Michael additions can also be mediated by catalysts that rely on hydrogen‐bonding to control reactivity and stereocontrol.^[108]^ These catalysts are particularly interesting under ball‐milling conditions because solvents can stabilise the hydrogen‐bonding network, and hence it may be expected that the performance of these catalysts would suffer under stirred neat or ball‐milling conditions. Xu and co‐workers found that the Michael addition of various 2,4‐dicarbonyls (**11**) to nitroalkenes (**9**) could be effectively carried out in a ball mill, catalysed by cinchona alkaloid‐derived squaramide (**C11**), in as little as 5 min with 0.5 mol% catalyst loading (Scheme 6).^[136]^ For the reaction between acetyl acetone (**11** **a**) and β‐nitrostyrene (**9** **a**), the desired product (**12** **a**) was furnished in high yield and enantioselectivity for the ball‐milled process. An alternative report details a stirred neat comparator, although in this instance a fullerene‐based thiourea catalyst (**C12**) was employed and afforded the product in 87 % yield and 84 % *ee* after 4 h (Scheme 6B).^[137]^ The authors reported a solution comparison, conducted in dichloromethane (DCM), and catalysed by squaramide (**C11**), which gave comparable yield and enantioselectivity, albeit in a much longer reaction time of 8 h. This report demonstrates that not only can ball‐milling compete with stirred solution methods in terms of yield and stereoselectivity, in hydrogen‐bonding‐mediated organocatalysis; it does so with much‐reduced reaction times.

**Scheme 7:**
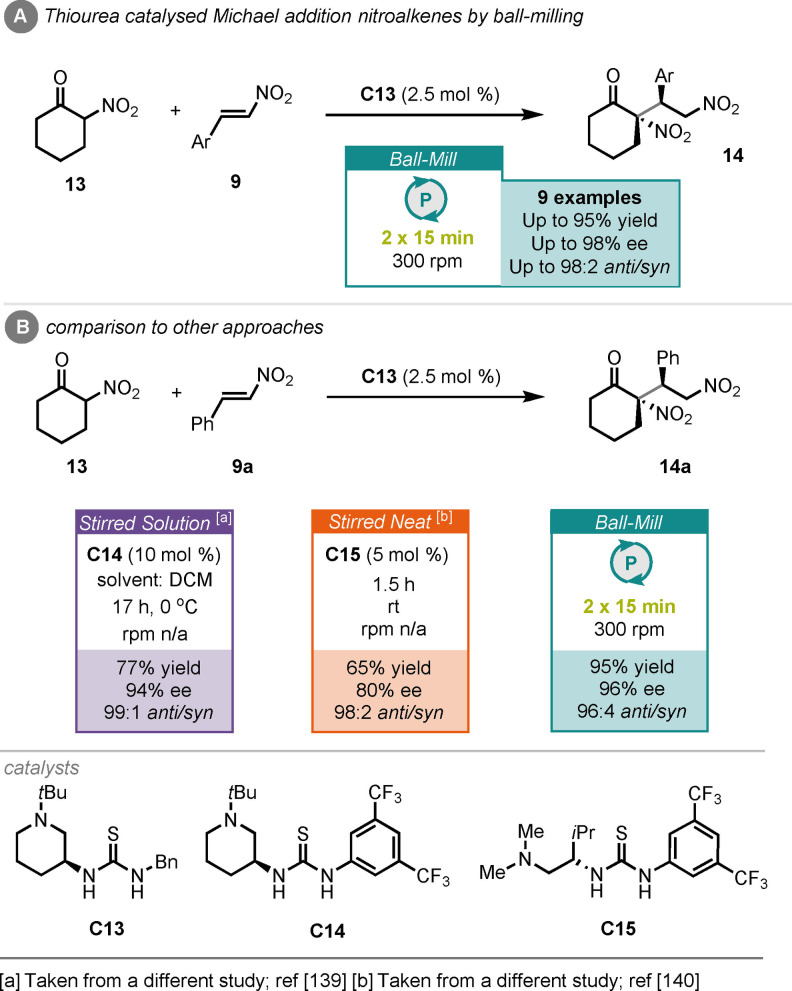
(A) Thiourea‐catalysed Michael addition of α‐nitrocyclohexanone to nitroalkenes by ball‐milling. (B) Comparison to other approaches.

Following on from this, Bolm and co‐workers reported the thiourea (**C13**)‐catalysed Michael addition of α‐nitrocyclohexanone (**13**) to various nitroalkenes (**9**), under ball‐milling conditions (Scheme 7A).^[138]^ With optimized conditions Michael addition products (**14**) could be accessed in up to 95 % yield, 98 % *ee* and 98 : 2 *anti*/*syn* ratio, in as little as 30 min. This was a huge improvement over their previous work under stirred solution conditions, where reaction times of 17 h were required to obtain comparable results, albeit with a different thiourea catalyst **C14** (Scheme 7B).^[139]^ Furthermore, a previous report, using solvent‐free stirred neat conditions, required 1.5 h to achieve lower yield and stereoselectivity, albeit using a different thiourea catalyst (**C15**).^[140]^

**Scheme 8:**
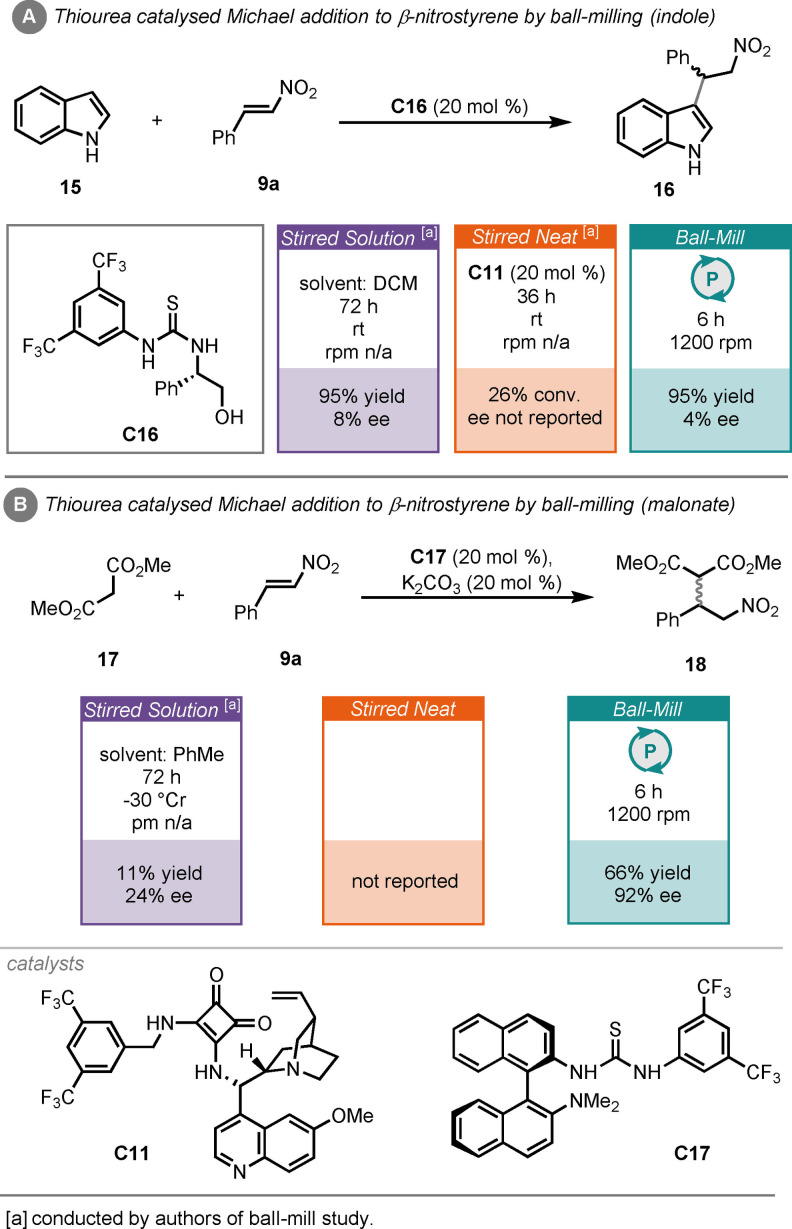
(A) Thiourea‐catalysed Michael addition of indole to β‐nitrostyrene by ball‐milling, with comparisons to other reactors. (B) Thiourea‐catalysed Michael addition of dimethyl malonate to β‐nitrostyrene by ball‐milling, with comparisons to other approaches.

Hestericová and Šebesta demonstrated that a variety of thiourea catalysts could mediate the Michael addition of indole (**15**) or dimethyl malonate (**17**) to β‐nitrostyrene (**9** **a**), under ball‐milling conditions (Scheme 8).^[141]^ Thiourea catalyst (**C16**) was found to be particularly effective for the reaction between indole and β‐nitrostyrene, giving the product (**16**) in 95 % yield in just 6 h, although this reaction proceeded with poor stereocontrol (Scheme 8A). However, when the same catalyst was used under stirred solution (DCM) conditions, a reaction time of 72 h was required to achieve comparable results, including poor stereoselectivity. An alternative squaramide catalyst (**C11**), was required to enable the reaction under neat conditions and led to poor conversion after 36 h. A similar outcome was observed for the reaction between dimethyl malonate and β‐nitrostyrene, where BINAM‐based thiourea catalyst (**C17**) was found to be most effective at catalysing the reaction, along with potassium carbonate as a base, giving the product (**18**) in 66 % yield and 92 % *ee*. This reaction has not been reported under solvent‐free stirred conditions; however, under solution conditions (DCM) a reaction time of 72 h was required to obtain the product in poor yield and enantioselectivity (Scheme 8B). This report, again, demonstrates the possibilities of improving on enantioselectivites by conducting organocatalysis under ball‐milling conditions.

**Scheme 9:**
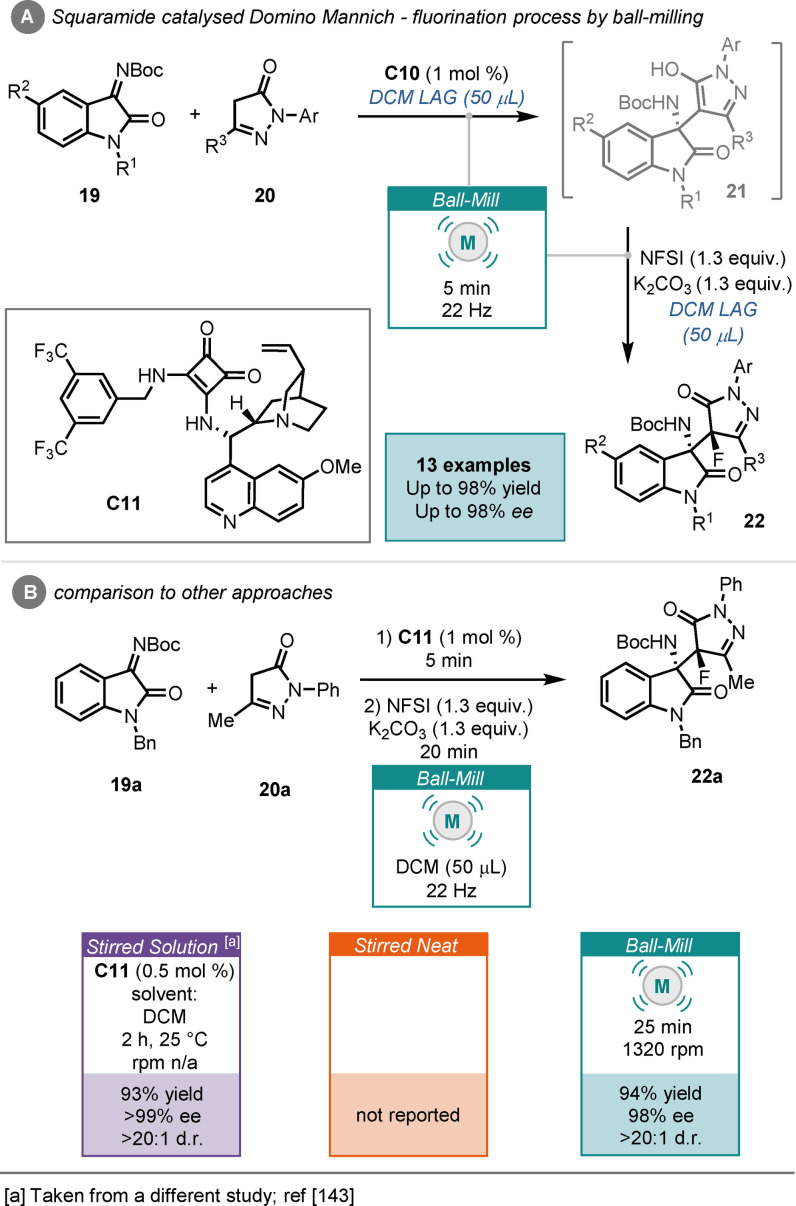
(A) Squaramide‐catalysed domino Mannich‐fluorination reaction by ball‐milling. (B) Comparison to other approaches.

Recently, Šebesta and co‐workers reported an asymmetric domino Mannich‐fluorination process, catalysed by squaramide (**C11**) under ball‐milling conditions.^[142]^ Their work involved the reaction between isatin derived ketimines (**19**) and pyrazolones (**20**), followed by fluorination using *N*‐fluorobenzenesulfonamide (NFSI) to yield products (**22**) in as little as 25 min, with good to excellent yields and excellent stereocontrol (Scheme 9). They utilized DCM as a LAG agent in this work, which was shown to improve the reaction, in terms of product yield and stereocontrol. No solution or neat stirred examples were carried out, however; a previous report in solution demonstrated that the reaction between ketimine (**19** **a**) and pyrazolone (**20** **a**) could be complete in 2 h, with similar yields and stereocontrol observed to the ball‐milled process (Scheme 9B).^[143]^

## 3 Tertiary Amine Organocatalysis

### 3.1 Morita‐Baylis‐Hillman reaction under ball‐milling conditions

**Scheme 10:**
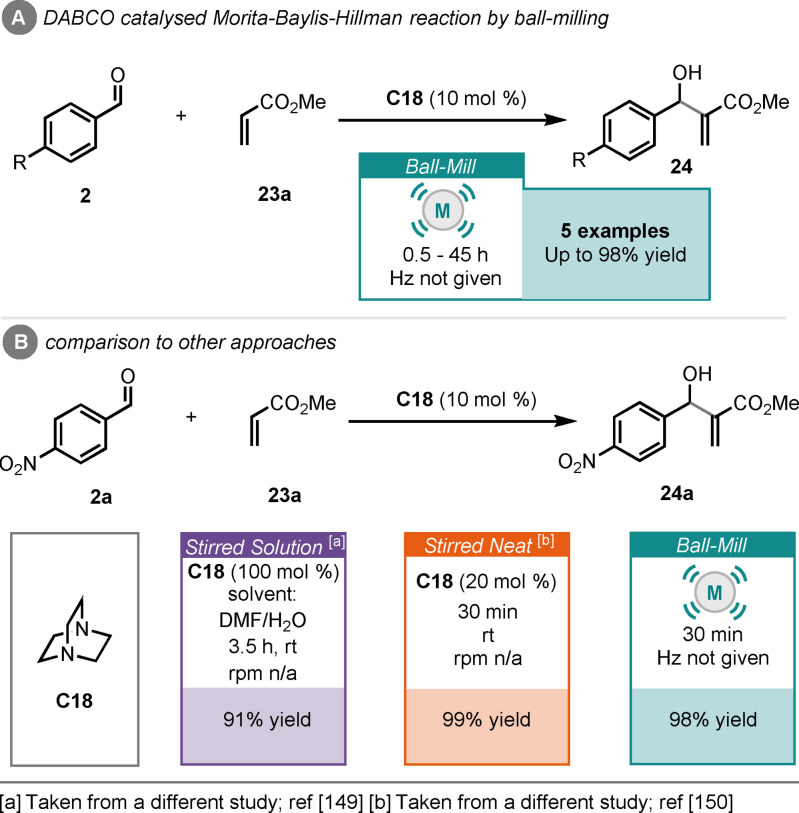
(A) Tertiary amine (DABCO)‐catalysed MBH reaction by ball‐milling. (B) comparison to other approaches.

The Morita‐Baylis‐Hillman (MBH) reaction is a well‐established and powerful C−C bond forming reaction, typically between aldehydes and α,β‐unsaturated compounds to produce functionalised allylic alcohols.[^144^, ^145^] The MBH reaction can be catalysed by tertiary phosphine or tertiary amine organocatalysts, with a plethora of examples, including asymmetric versions, in the literature over the past few decades.[^146^, ^147^] However, the MBH reaction can be very slow (days) and typically uses toxic solvents such as DCM or THF. To rectify this, Mack and Shumba reported a ball‐milling‐enabled MBH reaction between aryl aldehydes (**2**) and methyl acrylate (**23** **a**), catalysed by diazabicyclo[2.2.2]octane (DABCO, **C18**). They were able to access the products (**24**) in up to 98 % yield, in as little as 30 min when *p*‐nitrobenzaldehyde was used (Scheme 10).^[148]^ This was much faster than a report of the reaction under solution conditions (DMF/water mix), where a reaction time of 3.5 h was required to achieve comparable results using stoichiometric DABCO.^[149]^ The stirred neat reaction gave the product (**23** **a**), after 30 min, in equal yield to the ball‐milling approach but with twice the catalyst loading.^[150]^ Notably, no asymmetric versions of this reaction are reported under milling conditions.

**Scheme 11:**
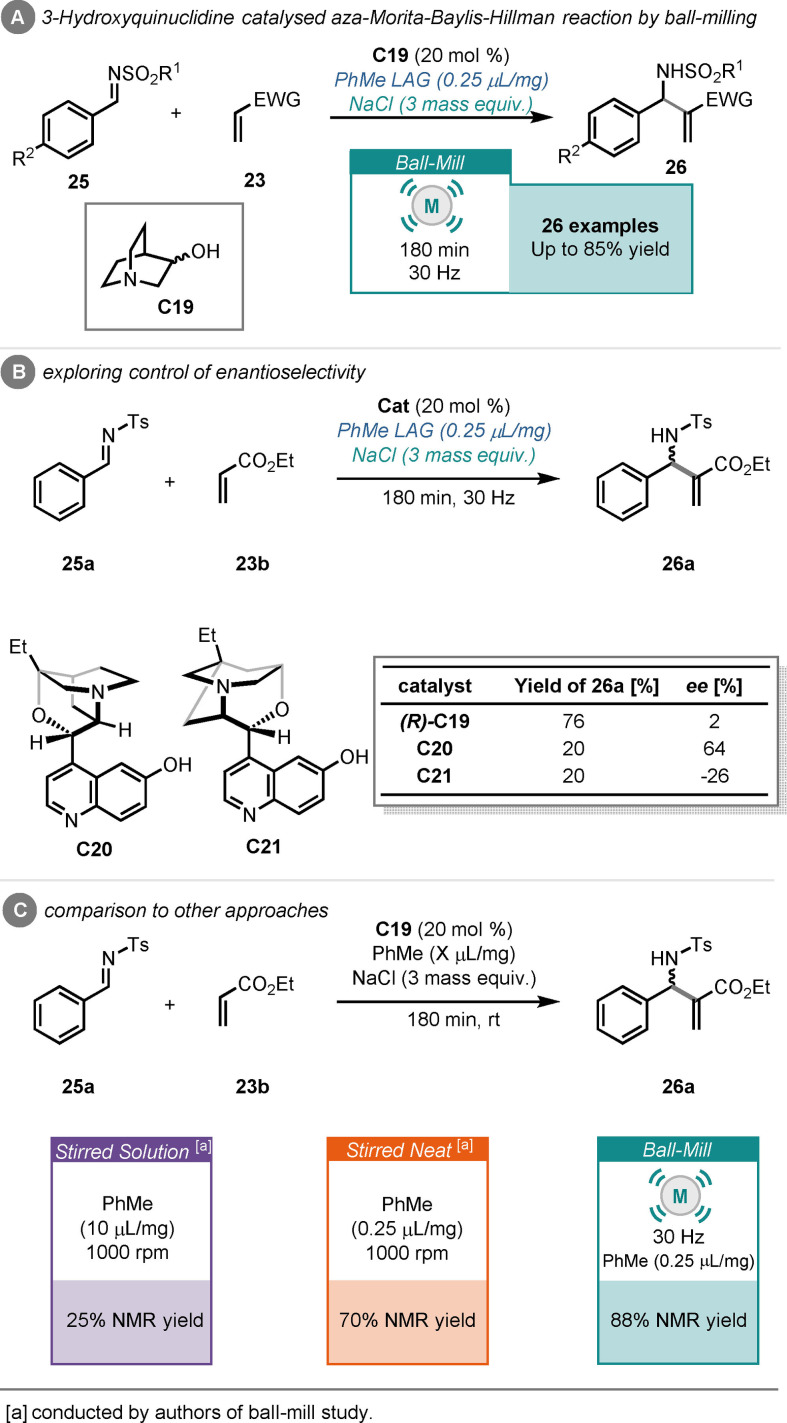
(A) 3‐hydroxyquinuclidine‐catalysed aza‐MBH reaction between imines and Michael acceptors by ball‐milling. (B) Investigations into enantioselective control. (C) Comparisons to other approaches.

Inspired by the work of Mack and Shumba, Browne and co‐workers recently reported an aza‐MBH reaction under ball‐milling conditions, that is, reaction between imines (**25**) and α,β‐unsaturated compounds (**23**).^[151]^ It was demonstrated that 3‐hydroxyquinuclidine (**C19**) could effectively catalyse the reaction in as little as 99 min, using toluene as an additive in LAG quantities and sodium chloride as a grinding auxiliary and furnishing the desired products (**26**) in moderate to excellent yield (Scheme 11). It was also shown that good enantiocontrol could be imparted by utilizing β‐isocupreidine (**C20**) as catalyst: under the previously optimized ball‐milling conditions, 64 % *ee* was obtained, albeit in low yield (Scheme 11B). Finally, comparisons to solution‐stirred and neat‐stirred approaches were carried out, effectively demonstrating that the solution analogue is much slower than the ball‐milled process, as only 25 % NMR yield of product was observed after 3 h. Likewise, the dry‐stirring analogue was inferior to the ball‐milled approach, providing 70 % NMR yield of product after 3 h (Scheme 11C).

## 4 Acyl Anion Reactions Under Ball‐Milling Conditions

**Scheme 12:**
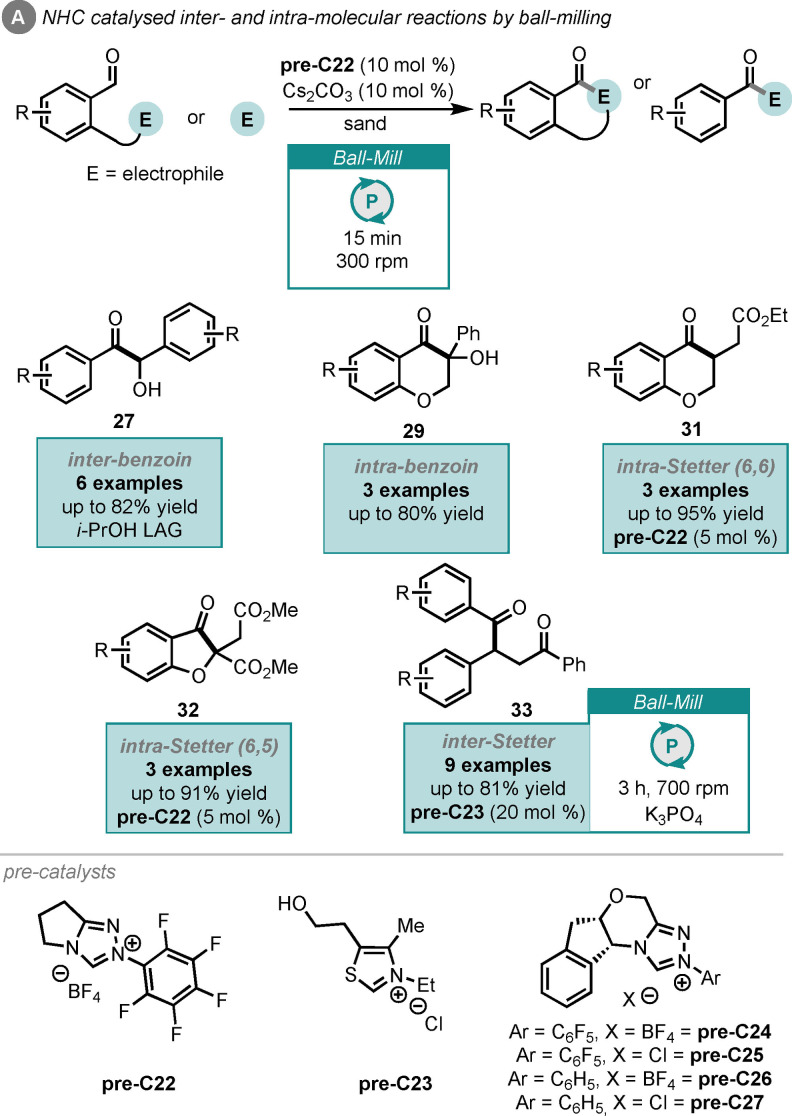
NHC‐catalysed inter‐ and intramolecular benzoin and Stetter reactions by ball‐milling.

Acyl anions are a class of activated carbonyls, whereby *umpolung* reactivity is invoked, opening the possibility for functionalisation, which was previously inaccessible. This reactivity can be accessed using N‐heterocyclic carbenes (NHCs) and was pioneered by work from Breslow.^[152]^ Two of the transformations possible using acyl anion chemistry are benzoin and Stetter reactions, where benzaldehyde derivatives react with carbonyls or α,β‐unsaturated carbonyls, respectively. Like the previous transformations in this Review, benzoin and Stetter reactions are well studied under both stirred conditions with and without solvent.^[109]^ Recently, Browne and co‐workers reported the first acyl anion NHC organocatalysis under ball‐milling conditions (Scheme 12A).^[153]^ Their work included inter‐ and intramolecular benzoin and Stetter reactions, producing products **27**, **29** and **31**–**33**, and demonstrated a rate enhancement over solution‐phase reports. This work utilized both a grinding auxiliary (sand in this case) and a LAG agent.^[34]^ The remaining reaction conditions were the use of triazolium or thiazolium pre‐NHC catalysts (**pre‐22** to **pre‐C27**) and caesium carbonate as a base.

**Scheme 13:**
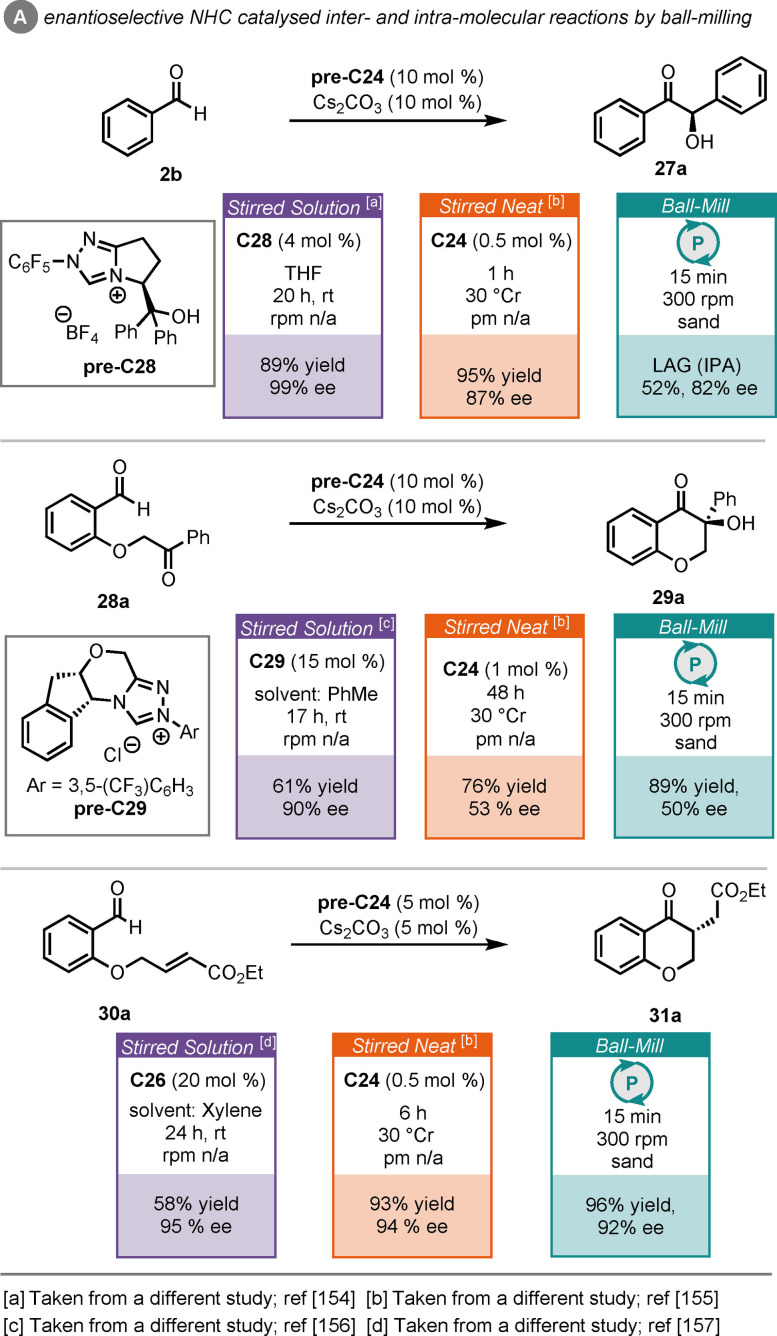
Comparison of ball‐milled benzoin and Stetter reactions to other approaches.

This work included some enantioselective examples, also, catalysed by chiral **pre‐NHC 24**; with the intermolecular benzoin condensation of **2** **a**, the intramolecular benzoin condensation of **28** **a**, and the intramolecular Stetter reaction of **30** **a** included in their studies (Scheme 13A). Up to 92 % *ee* was achieved here, which compares well with the results that have been obtained in solution stirred and neat stirred reports, with the added benefit of significantly reduced reaction times.[^154^, ^155^, ^156^, ^157^]

## 5 Other Transformations Under Ball‐Milling Conditions

**Scheme 14:**
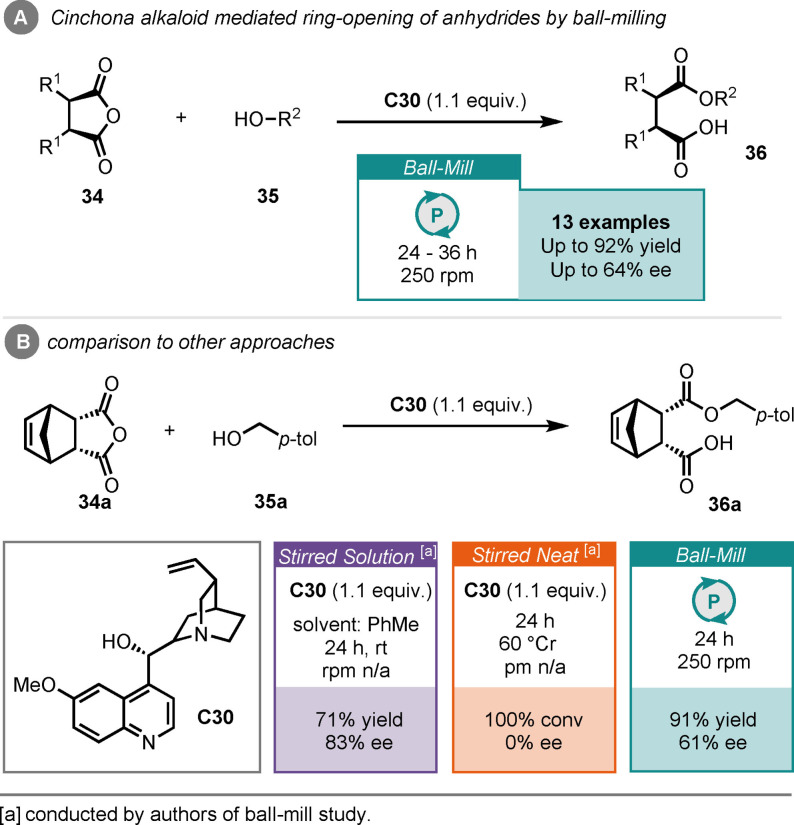
(A) Cinchona alkaloid‐mediated ring‐opening of cyclic anhydrides by ball‐milling. (B) Comparison to other approaches.

While most of the ball‐milling enabled organocatalysis has focused on secondary amine or hydrogen‐bonding catalysts, there are a few reports that do not fit these descriptors. One such report is that of a desymmetrisation process by Bolm and co‐workers, where cyclic anhydrides (**34**) underwent ring‐opening in the presence of an alcohol (**35**), mediated by the cinchona alkaloid quinidine (**C30**), in a ball‐mill (Scheme 14A).^[158]^ This was one of the first reports of a ball‐milling process being mediated by an organic compound, although quinidine was used in superstoichiometric quantities; hence, this is not an organocatalytic process per se but does represent an exciting desymmeterisation. Using this methodology, they were able to access a variety of ring‐opened anhydrides (**36**) in good yields and with moderate enantiocontrol. For the reaction between anhydride **34** **a** and *p*‐methylbenzyl alcohol **35** **a**, they obtained ring‐opened product **33** **a** in 91 % yield and 61 % *ee*, after 24 h of milling, which was carried out by breaking up 25 min milling cycles with 5 min rest periods (Scheme 14B). This milling format, with programmed regular pauses, was implemented to minimise overheating of the milling jars, which was found to lead to reduced enantiocontrol. When compared to stirred methods, also reported in the original paper, it was found that the solution reaction performed slightly better, in terms of enantioselectivity, after the same reaction time. The stirred neat reaction, on the other hand, required harsher conditions (60 °C) to reach full conversion; this led to a racemate being formed. This very early report showed ball‐milling had potential in organic transformations, particularly at enabling the solvent‐free reaction, but control of the specific parameters is imperative to achieve good enantiocontrol.

**Scheme 15:**
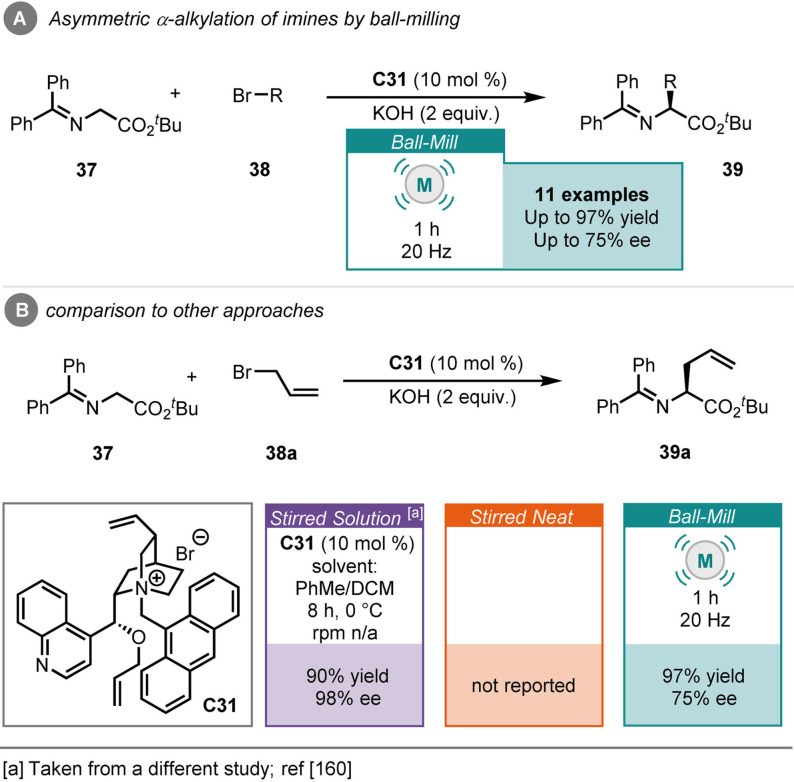
(A) Asymmetric α‐alkylation of imines by ball‐milling. (B) Comparison to other approaches.

Lamaty and co‐workers reported the asymmetric α‐alkylation of imines (**37**) with alkyl bromides (**38**), catalysed by cinchonidine‐derived ammonium salt (**C31**) and potassium hydroxide as a base, under ball‐milling conditions (Scheme 15A).^[159]^ The products (**39**) were obtained in good yields and with moderate enantiocontrol, after 1 h of milling. This process is of particular interest as it delivers non‐proteinogenic α‐amino acids. No stirred neat comparisons have been reported; however, a previous report demonstrates that the solution reaction (described as phase transfer) requires up to 8 h to achieve comparable yields, but with much greater enantiocontrol (Scheme 15B).^[160]^

## 6 Conclusions and Reflections

This Review summarises the combination of organocatalysis with ball‐milling techniques and specifically looks at comparing the performance of ball‐milling methods versus conventional stirring with and without solvent, with a focus on the difference in reaction time, yield and any relevant stereocontrol. It is clear that ball‐milling mechanochemistry can offer significant rate enhancements in many cases under solvent‐free conditions, without necessarily sacrificing performance. Returning to the original proposed question, *do high enantioselectivities from milled reactions contradict what you might expect?* The data presented does not lead to a strong answer to this question. Certainly, examples of high enantioselectivities are achievable by milling, but so too are selectivities that are not as high as solvent comparators.^[161]^ There are many factors to consider, and some of our thoughts are summarised here. Reporting of low enantiomeric excesses (*ee* values) as part of an optimised process is likely to be underrepresented in the literature, meaning that the data presented is inherently biased. The energy of each collision in a ball mill results in an instantaneous and short‐lived temperature and pressure increase (volume reduction), and in some cases the temperature is not dissipated rapidly and the jar itself begins to warm. This increase in bulk temperature during milling is infrequently reported and quite challenging to measure in practice. Bulk temperature increases could be at odds with trying to achieve high enantioselectivities in some cases; indeed, the use of intermittent pauses in some milled reaction examples highlights this trade‐off. Enantioinduction is often a result of relative rates of the stereocontrolled process versus the background stereo‐uncontrolled process. Both the rate of the controlled and uncontrolled processes can be affected in differing ways by temperature and volume changes. Certainly, a series of systematic studies on how milling parameters effect *ee* values could help to shed more light on this important fundamental question. By and large this Review has demonstrated that neat stirring is typically (though not always) a less effective method to conduct organocatalytic reactions; this could be attributable to poor mass transfer (mixing) as compared to solution and milled techniques.

In summary, we have surveyed and discussed mechanochemical organocatalysis, an area that remains in its relative infancy, with many catalyst types and activation modes still being little or completely unexplored. These include chiral phosphoric acids, tertiary amines and phosphines, and N‐heterocyclic carbenes. There appears to be no general trend when comparing milled versus solution for covalent and non‐covalent organocatalytic modes. However, if the solution reaction requires sub‐ambient temperatures to achieve good enantioselectivity, it appears these reactions do not fare well in a ball mill. Thus, over the next five to ten years, we fully expect to see this area expand further and develop an increasing understanding of stereocontrol by milling conditions.

## Conflict of interest

The authors declare no conflict of interest.

## Biographical Information

*Matthew T. J. Williams received his M.Chem. degree with a year abroad at the University of Florida from Cardiff University in 2018. He conducted his final year research project under the guidance of Dr Louis Morrill. Then, he began his Ph.D. studies at Cardiff University in October 2019 under the joint guidance of Louis Morrill and Duncan Browne, as part of the Centre for Doctoral Training in catalysis Ph.D. programme. His research focuses on utilising enabling technologies for synthesis and catalysis*.

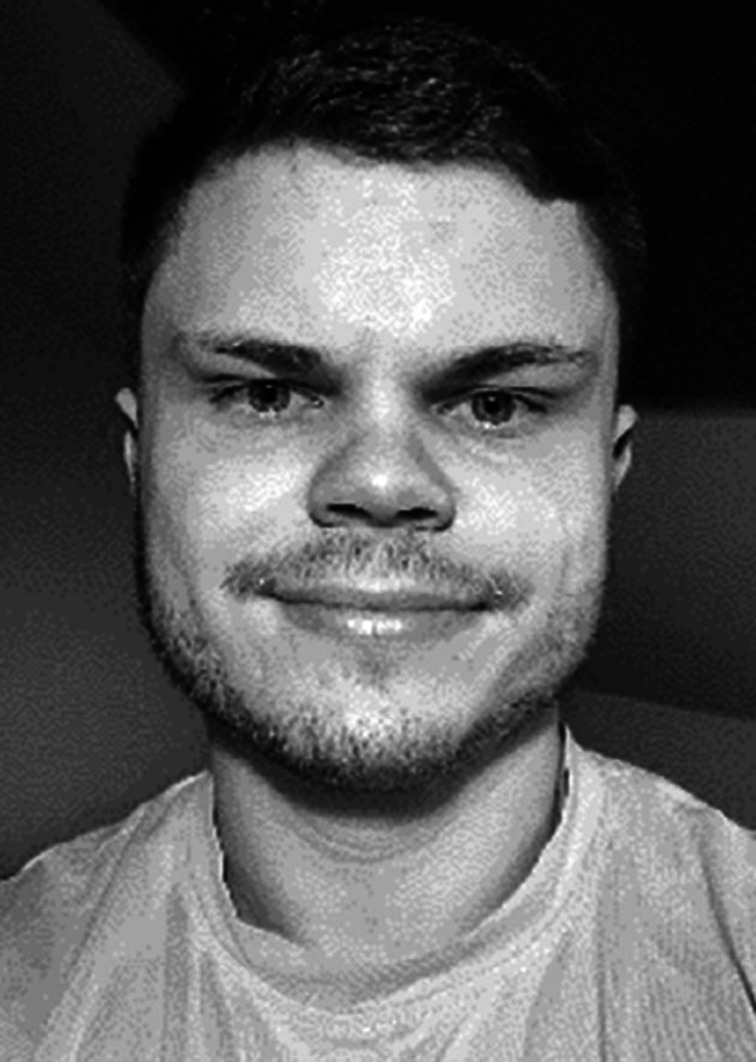

## Biographical Information

*Louis C. Morrill received his Ph.D. from the University of St Andrews in 2014 under the direction of Prof. Andrew Smith and undertook postdoctoral research at UC Berkeley with Prof. Richmond Sarpong. In June 2015, he initiated his independent research career at Cardiff University. Research in the group is focused on inventing new reactions in organic chemistry and developing sustainable catalytic methodologies for synthesis*.

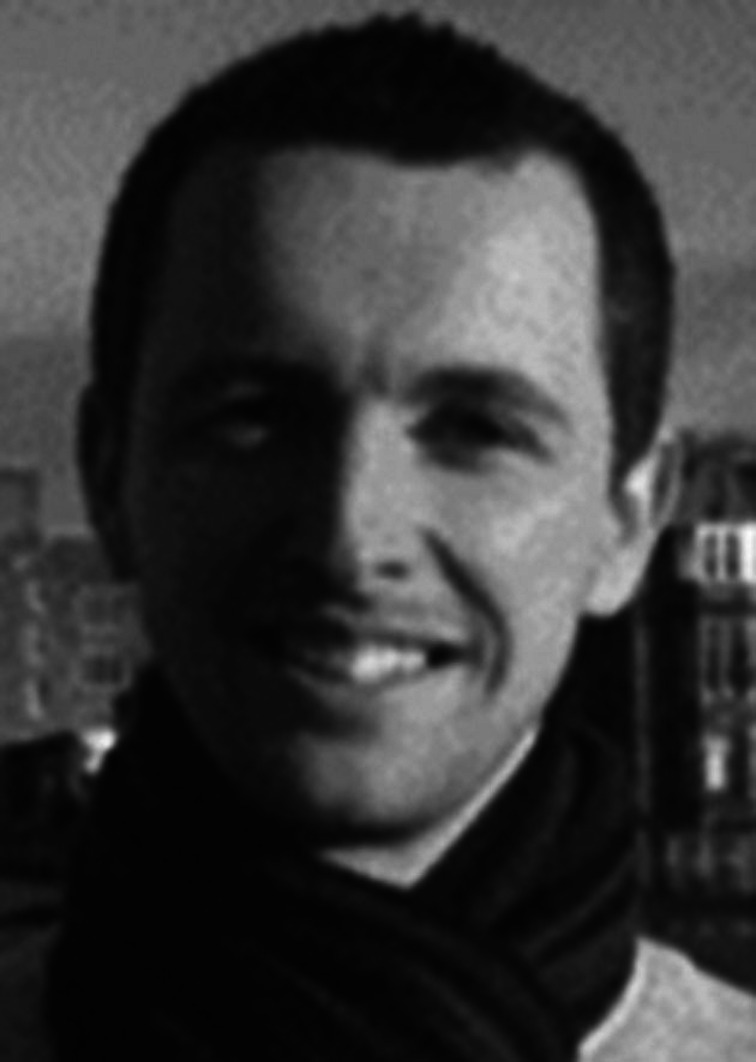

## Biographical Information

*Duncan L. Browne received his Ph.D. in organic synthesis from the University of Sheffield under the supervision of Prof. Joseph P. A. Harrity (Syngenta, CASE). This was followed with a one‐year Doctoral Prize Fellowship from the EPSRC before moving in 2010 to the University of Cambridge for postdoctoral studies with Professor Steven V. Ley FRS CBE. In September 2014, Duncan established his independent research group at Cardiff University focussing on the use of enabling technologies for synthesis and catalysis. In 2019 he was recognised by both Green Chemistry and Reaction Chemistry and Engineering as an “Emerging Investigator” and was a recipient of a Thieme Chemistry Journal Award. In 2019, he was appointed as Associate Professor of Organic Synthesis and Drug Discovery at UCL (University College London)*.